# Quality of Life for Adults with Prader–Willi Syndrome in Residential Group Homes

**DOI:** 10.3390/jcm13113323

**Published:** 2024-06-04

**Authors:** Hadassa Mastey Ben-Yehuda, Varda Gross-Tsur, Harry J. Hirsch, Larry Genstil, Dvorit Derei, Dorit Forer, Fortu Benarroch

**Affiliations:** 1 Israel National Multidisciplinary Prader-Willi Syndrome Clinic, Department of Neuropediatrics, Shaare Zedek Medical Center, Jerusalem 9103102, Israel; hadassamastey@gmail.com (H.M.B.-Y.); varda.gross@gmail.com (V.G.-T.); hirschmd@gmail.com (H.J.H.); genstil@gmail.com (L.G.); ddvorit@gmail.com (D.D.); fdorit6@gmail.com (D.F.); 2 The Faculty of Medicine, The Hebrew University School of Medicine, Jerusalem 9112002, Israel; 3 Herman Dana Division of Child and Adolescent Psychiatry, Hadassah-Hebrew University Medical Center, Jerusalem 9112001, Israel

**Keywords:** Prader–Willi syndrome, quality of life, group homes

## Abstract

**Background**: Strict regimens of restricted caloric intake and daily physical exercise are life-saving in Prader–Willi syndrome (PWS) but are extremely challenging in home environments. PWS-specialized hostels (SH) succeed in preventing morbid obesity and in coping with behavioral disorders; however, effects of restricted living environments on quality of life (QOL) have not been described. Evidence on QOL is critical for clinicians involved in placement decisions. **Methods**: We examined the impact of living in SH versus at home or in non-specialized hostels (H and NSH) on QOL, behavior, and health parameters. All 58 adults (26 males) followed-up in the National Multidisciplinary Clinic for PWS were included: 33 resided in SH, 18 lived at home, and 7 lived in NSH. Questionnaires were administered to primary caregivers to measure QOL, and data were obtained from the medical records. **Results**: The H and NSH group were compared with those for adults in SH. Despite strict diet and exercise regimens, QOL was similar for both groups. Eight-year follow-up showed that food-seeking behavior decreased in SH but increased in H and NSH. BMI, cholesterol, and triglyceride levels were lower in SH. **Conclusions**: Our results suggest that living in SH is associated with benefits for physical health and behavior without negatively affecting QOL.

## 1. Introduction

Prader–Willi Syndrome (PWS) is a complex neurogenetic disorder due to lack of expression of imprinted genes in the q11–q13 region of paternally inherited copies of chromosome 15. Genetic subtypes include deletions of the critical region on chromosome 15, maternal uniparental disomy, and, rarely, defects in the imprinting center. Hypotonia, feeding difficulties, and delayed developmental milestones are typical manifestations in infancy. Later in childhood, hyperphagia and lack of satiety result in excessive weight gain, leading to morbid obesity unless strict compliance with diet and exercised regimens are maintained. Other common features of PWS include behavioral and psychiatric disorders, variable degrees of cognitive disability, growth hormone deficiency, hypogonadism, and skin picking [1,2,3].

Psychiatric disorders and behavioral problems become more prevalent with increasing age, along with a decline in functional, behavioral, and cognitive abilities [4,5,6]. The prevalence of obesity, the leading cause of morbidity and mortality in PWS, increases from 40% in younger individuals to 82–98% in adults [2,7,8,9]. While obesity is a major cause of morbidity and mortality in PWS, even individuals with a normal BMI have a higher-than-average incidence of medical problems such as sleep disorders, skin picking, swallowing dysfunction, and abnormal gastric motility.

Life expectancy of individuals with PWS has been increasing due to improved awareness of maintaining strict adherence to dietary restrictions, “food security”, and exercise programs along with early initiation of growth hormone treatment [10]. The 24 h daily supervision needed to control the hyperphagia and behavioral disorders presents new challenges for families of adolescents and adults. Many families are unable to cope with the emotional and physical demands for these individuals. In addition, the need for constant supervision is a financial burden for parents who need to remain at home. As an alternative to home care, residential group homes dedicated specifically to the care of individuals with PWS provide an environment with “food security”, enforce strict adherence to diet and exercise regimens, maintain rigid guidelines for managing behavioral disorders, and closely monitor health issues. Adults with PWS who live in dedicated group homes achieve and maintain significantly lower BMI values compared to age-matched controls living with their families at home: BMI (mean ± SD) decreased from 36.3 ± 11.0 kg/m^2^ upon admission to 27.0 ± 5.6 kg/m^2^ after seven years. The mean BMI was significantly lower in the group home residents (27.9 ± 7.1 kg/m^2^) compared to age-matched individuals living at home (36.8 ± 12.7 kg/m^2^, *p* = 0.008) [11]. A recent survey of pooled international archival data showed that in people with PWS, there is a significant reduction in weight and BMI after joining a full-time care service, but the results were not compared with those of people with PWS living at home [12].

Despite the proven health benefits of group home living, parents are often concerned about the effects of the highly structured environment on quality of life (QOL). There are, to date, only two studies addressing QOL in adults with PWS, one of which discussed the impact of weight and dysmorphic features on QOL, while the other addressed the influence of psychiatric disorders on QOL [13,14]. Neither of these reports compared the effect of residing in dedicated group homes on QOL with that of living in other settings. In the current study, we compared health, behavior, and overall QOL for adults with PWS living in PWS-specific hostels (SH) with those individuals residing at home with their families (H) or in non-specialized hostel settings (NSH).

## 2. Methods

### 2.1. Study Population

Nearly all individuals in the country with a confirmed diagnosis of PWS are followed-up annually at the national multi-disciplinary clinic of our Medical Center. Of the 73 PWS adults older than 18 years (age range 18–56 years) followed-up in our clinic, 34 lived in one of the two specialized hostels (SH) in the country for adolescents and adults with PWS, 30 lived at home (H), and 9 were in non-specialized residential homes. A short description of the PWS-specialized group homes in the country can be found in Appendix A.1.

The inclusion criteria for the study are adults over 18 years of age and who are followed-up regularly in the multi-disciplinary clinic. The data were collected in 2021. Fifteen adults were excluded from the study: eight individuals who did not regularly attend the multidisciplinary clinic, four individuals living at home whose parents were not fluent enough in the language to understand the questionnaires, two young adults who refused to participate in the study, and a 21-year-old from the home group who died during the research period. The research group included 58 adults, of whom 33 lived in SH, 18 at home, and 7 in NSH.

The study was approved by the institutional review board of the Medical Center. Informed consent was signed by either the individuals with PWS or their guardians.

### 2.2. Study Design

1. The primary caregivers were asked to complete all three of the following questionnaires:
  - Quality of Life Questionnaire—Short-Form Survey (SF-36) is a tool for assessing health-related functioning and quality of life [15]. The questionnaire consists of 36 questions designed to evaluate eight health domains: (1) “Physical Functioning”—limitations in physical activities due to health problems, (2) “Role—Physical”—limitations in usual role activities due to physical health problems, (3) “Bodily Pain”, (4) “General Health”—perception of overall health, (5) “Role–Emotional”—limitations in usual role activities due to emotional problems, (6) “Vitality”—energy and fatigue, (7) “Mental Health”—overall mental health (psychological distress and psychological well-being) and (8) “Social Functioning”—limitations in social activities due to physical or emotional problems. Each of the domains receives a score in the range of 0–100. A higher score indicates better health-related functioning and quality of life. The validation study of the Hebrew version of the SF36 questionnaire in adults found Cronbach’s alpha coefficients ranging from 0.76 to 0.93. Thus, the scores for all scales met the customary level of scale reliability [15]. In this validation study, professionals trained for this purpose collected information from the respondents and filled out the questionnaire based on their judgment. This approach is quite similar to what has been implemented in our study: The parents or caretakers, who were instructed on how to use the questionnaire, completed it based on their thorough familiarity with the respondents.
  - Prader–Willi Syndrome Behavioral Questionnaire (PWSBQ) is a questionnaire filled out by parents or caregivers for clinical monitoring and research purposes, which aims to define the behavioral profile characteristics of each participant [16]. The questionnaire includes 30 questions divided into four domains referring to common behavioral patterns in PWS: abnormal emotional regulation, food-seeking related behavior, lack of flexibility and oppositional behavior, and interpersonal problems. Each domain includes several statements describing common PWS behaviors, which are rated on a 5-point scale, from 0 (not true) to 4 (extremely true). A higher score represents a more disturbed behavior. All items on the PWSBQ were analyzed for internal reliability by calculating Cronbach’s alpha, which ranged from 0.633 to 0.870. The results were found to be reliable.
  - Waisman Activities of Daily Living Scale (W-ADL) is a measure of daily functioning for adolescents and adults with disabilities. Across the disability groups, the WADL questionnaire was found to be reliable, with Cronbach’s alphas ranging from 0.88 to 0.94 [17]. The scale, consisting of 17 items, is administered to caregivers and assesses the level of functioning and independence of the individual. Each item is rated on a scale of 0–2, with 0 representing inability. The scoring range is 0–34, with 34 indicating full independence.
2. Medical information extracted from the patients’ medical records at the multidisciplinary clinic included the following:
  - Demographic data (age, gender, genetic subtype), medical diagnoses, and medications;
  - Repeated measurements of height, weight, BMI, and bone density tests (DEXA SCAN);
  - Laboratory tests performed as part of the routine clinical monitoring, including complete blood count, fasting glucose, hemoglobin A1c, lipid profile, liver and renal function tests, free T4, TSH, and vitamin D levels.
3. Information was also extracted from the SF-36 and PWSBQ forms, which were filled out by caregivers for the 53 participants in 2013 for a previous study [13]. Four participants who changed their living conditions between the two measurements were excluded. We compared results from the 2013 study with the results obtained in 2021 for each individual;
4. Statistical analysis of the data was conducted using Python 3.8 software and the robust linear regression (RLR) model. Analysis of data from the questionnaires considered potential confounders such as age, height, sex, genetic profile, growth hormone treatment, and duration of growth hormone treatment. For the repeated measurements of BMI, blood tests, and bone density measures, generalized estimating equations (GEE) analysis was employed.

## 3. Results

### 3.1. Characteristics of the Study Participants

Table 1 shows the demographic data for participants living in SH compared with those individuals living at home or in non-specialized group homes (H and NSH).

### 3.2. Questionnaire Analysis

#### 3.2.1. Quality of Life Questionnaire (QOL)

As shown in Table 2, QOL scores on the SF-36 questionnaire showed higher physical functioning for the SH group compared to H and NSH but similar results for other domains. General health scores were slightly lower for SH. Analysis of questionnaire scores from 2013 to 2021 revealed no significant changes in average scores across different domains (Table A1 in the Appendix A.2 shows the full parameters comparison).

#### 3.2.2. Prader-Willi Syndrome Behavioral Questionnaire (PWSBQ)

There is an intrinsic bias since individuals who are sent to SH are those with more oppositional behavior and interpersonal problems compared to the H&NHS group as shown in Table 3. Nevertheless, PWSBQ showed fewer food-seeking behaviors in the SH group.

Upon comparing PWSBQ scores from 2013 to those of the present study, a significant change was observed in the “Food-seeking-related behavior” domain. For the SH group, there was a significant reduction in these behaviors by 41.6%, while the H and NSH group experienced an increase of 22.8%. This represented a total change difference of 64% (*p* = 0.003) between the two groups (Table A2 in the Appendix A.3 shows the full parameters comparison).

#### 3.2.3. Waisman Activities of Daily Living Scale (W-ADL)

No significant difference was found between the research groups in the W-ADL questionnaire. The W-ADL score was 21.2 ± 4.8 for the SH group and 18.7 ± 6.8 for the H and NSH group (*p* = 0.21).

### 3.3. Repeated BMI Measurements

In the present study, repeated measurements of BMI taken over the follow-up years were collected for all 33 from the SH group and 25 adults living at H and NSH. The mean time between the first and last exams was 11.1 ± 4.2 years in the SH group (range, 0.7–21.6) versus 12.8 ± 6.9 years in the H and NSH group (range, 0.9–30.8).

The mean BMI of the SH group was lower by 5.73 kg/m^2^ than that of the H and NSH group (*p* < 0.001). Additionally, each additional year in the hostel lowered the mean by 0.57 (*p* < 0.001).

### 3.4. Medical Diagnoses

Five SH residents, ages 21 to 40 years, and three individuals in the H and NSH group, ages 15, 17, and 35 years, had diabetes mellitus. HbA1c values were lower in SH vs. H and NSH (7.0 ± 1.6% vs. 8.7 ± 1.3%). No differences in prevalence of hypothyroidism, scoliosis, or other medical conditions were found between the SH group and the H and NSH group (full list of medical diagnoses specified in Appendix A.4).

### 3.5. Comparison of Bone Density Measurements

Bone mineral density (BMD) in the lumbar vertebrae was higher for the SH group by 0.06 g/cm^2^ when standardizing for height and weight (*p* = 0.049) and by 0.1 g/cm^2^ when standardizing for BMI (*p* = 0.003). Bone density as measured at the hip was also greater in the SH by 0.05 g/cm^2^ when standardizing for height and weight (*p* = 0.040) and by 0.07 g/cm^2^ when standardizing for BMI (*p* < 0.001).

For each year of stay in a specialized hostel, the percentage increase in lumbar vertebral BMD was 7.57% when standardizing for height and weight (*p* = 0.044), 0.7% when standardizing for BMI (*p* = 0.012), and 0.69% without any standardization (*p* = 0.014). For each additional year spent in a specialized hostel, the odds ratio for having osteoporosis based on DEXA scans of the left hip was 1.62 times lower (*p* < 0.001) compared to the H and NSH group. The odds ratio for developing osteopenia was 1.84 times lower for SH vs. H and NSH (*p* < 0.001).

### 3.6. Laboratory Tests Measurements

All the parameters in the lipids’ profile were statistically significantly lower and vitamin D levels were higher in SH vs. H and NSH. A better average triglycerides/HDL cholesterol ratio was also observed (1.73 vs. 2.31, *p* = 0.000) in the SH group. No clinically significant differences were noted in other laboratory parameters (Table A3 in the Appendix A.5 shows the full parameters comparison).

## 4. Discussion

This study is the first comprehensive comparison of QOL, physical health, and behavior for adults with PWS living in specialized hostels compared with PWS adults living at home or in non-specialized group homes. The two groups in our sample were similar in demographic data except for the expected differences in mean age (slightly higher in the SH group) and in mean BMI (lower in the SH group). We found that QOL is similar in both groups. Furthermore, QOL does not decrease with long-term living in the specialized group homes. Difficulties in coping with severe behavior problems were common reasons for families to decide to transition individuals to a PWS-specific group home environment. Therefore, it is not surprising that we found higher scores on oppositional behavior among SH residents. Nevertheless, the 8-year follow-up showed a significant reduction in food-seeking behaviors in the SH group.

Because of the need for “food security” and to cope with characteristic behavior disorders of people with PWS, the hostels enforce a strict physical exercise regimen, caloric intake restriction, and assertive rules of conduct. Some parents are concerned that these policies and strict rules might have a negative impact on QOL. They claim that the staff in the SH may not relate to differences in personality traits and cognitive abilities among the various individuals with PWS and that living at home or in a less rigid hostel with a more heterogeneous population might allow better adaptation to the individual needs and personal abilities resulting in better QOL. Therefore, when parents are confronted with the decision whether or not to transition adolescents and adults with PWS to residential hostels, they wonder whether the improved physical health measures among SH residents may lead to a poorer QOL. We found that despite the strictly regulated behavioral environment, scores on the QOL questionnaires were not lower for the SH group compared to the H and NSH group. Compared with results of the same QOL questionnaire administered in 2013, the scores in the present study showed no significant change in QOL measures, indicating that long-term living in the designated group homes does not negatively impact QOL and that the significant improvements in health and behavior were not associated with deterioration in QOL.

One of the main factors in PWS that interferes with usual social activities is the constant occupation in food seeking, which is an integral part of the PWS behavioral phenotype, including hoarding or foraging for food, eating inedible objects, and stealing food or stealing money to buy food [18,19]. Despite the fact that the SH group is characterized by more severe behavioral problems, we found that in our sample, living in an SH led to a reduction in negative food-seeking behaviors, and compared with results from the 2013 study, we found a decrease of 41.5% in negative food-seeking behavior in the SH group versus an increase of 22.7% in the H and NSH group. Since the individuals in the SH group are less occupied with food seeking, they are more able to enjoy the social life that the SH offers. A recent survey of pooled international archival data also found an improvement in behavior after joining a full-time care service but, unlike our study, did not compare with people with PWS of the same age living at home. In addition, behavior was measured by a non-standardized tool, QOL was not measured, and the follow-up period was only a year [12].

The mean BMI in the SH group was 5.73 kg/m^2^ lower than for the H and NSH group, consistent with the findings of Dudley et al. [20] that living in an SH reduces the mean BMI by 3 kg/m^2^ compared to living at home with both parents and by 6 kg/m^2^ compared to living at home with a single parent. Furthermore, every year spent in a SH reduced the mean BMI by 0.57 kg/m^2^, as previously reported [11]. In the QOL questionnaire, the findings show better results for the SH group in the physical domain of “Physical Functioning” (limitations in physical activities due to health problems) and no significant differences in all the other domains except for “General Health” (a subjective perception of the caregiver about the general health status), most likely due to their older age and more consistent monitoring of health issues.

We also found that the SH group had lower cholesterol and triglyceride levels, higher vitamin D levels, and had a reduced risk of developing osteoporosis and osteopenia. There were no significant differences in other medical diagnoses compared with the H and NSH group, even though routine health surveillance including frequent laboratory tests was greater for the SH group. Earlier detection of medical conditions in the SH group may explain why other diagnoses were similar to findings in the H and NSH group, for whom other diagnoses may have been missed or delayed. Pellikaan et al. [21] reported that the frequency of missed medical diagnoses for Dutch adults with PWS living in SH was lower: In the SH group, 39% had missed one diagnosis, 9% missed at least two diagnoses, and 9% missed three diagnoses or more compared to 61%, 24%, and 9%, respectively, in the adults living at home [21]. Furthermore, the individuals living at home were younger than those in the SH group.

We want to emphasize that there are some very important positive effects of living in an SH that are not assessed by standardized QOL questionnaires. SHs provide a social environment appropriate for the cognitive disabilities and behavior disorders of adults with PWS, including a full schedule of organized group activities. Within the SH group, there is a greater possibility to arrange employment tailored to the unique needs of adults with PWS. Due to experience with specific needs for individuals with PWS, counselors in the SH are able to find appropriate workplaces and arrange for chaperoned transportation to the places of employment and close supervision throughout the working hours. In most cases, parents of PWS adults lack the experience and resources for arranging employment in a sheltered workplace with a secured food environment.

## 5. Limitations

The size of our sample limits the scope of the statistical analysis. Nevertheless, this is a nation-wide sample that includes nearly all adults in the country with this rare syndrome. Another limitation is that the measurements of behavior, functioning, and QOL were recorded by parents for those living at home and by caregivers (who are hostel professionals) for those living in hostels. Despite the training that we gave for all of the participants on how to fill out the questionnaires, the approach of the caregivers to the subjective assessment of behavior and QOL may be different from that of the parents, which might have caused some bias in the comparison. Caregivers may tend to judge their patients more strictly and may try to compare several individuals whom they are rating. By contrast, parents may tend to be more sympathetic, and each parent rates only his/her daughter/son. Published studies on the agreement between parents’ and staff’s ratings of QOL or behavioral problems actually revealed discrepancies in several but not all domains [22,23]. Interestedly, one of the findings was that clinical researchers may be more attuned to some externalizing behaviors compared to parents [23]. This observation may partly explain the observed phenomenon in our sample, where the SH group (assessed by group home staff) had higher mean score for oppositional behaviors compared to the H and NSH group (assessed by parents).

## 6. Conclusions

QOL is similar in adults with PWS who live in specialized hostels, at home, or in non-specialized group homes. Individuals in SH have fewer food-seeking-related behaviors, one of the main factors affecting the behavior and management of individuals with PWS. They also have lower BMI, even though they are older and have more oppositional behavior.

Our findings contradict the claim that the strict rules of SH negatively impact QOL. Furthermore, the benefits for social life and tailored employment are not apparent when evaluating the standardized QOL questionnaires. These findings may be helpful for families during the decision-making process for transitioning adolescents and adults with PWS to residential hostels. Further studies in different centers and countries are warranted in order to confirm these conclusions and to study potential cultural differences.

## Figures and Tables

**Table 1:** Characteristics of the study groups.

	PWS’s Specialized Hostels (SH) n = 33	Home or Non-Specialized Hostels (H and NSH) n = 25	*p*-Values
**Number of participants**	33	25	
**Females/males**	17/16	13/12	0.97 ^5^
**Age (years)** **(mean ± SD range)**	32.8 ± 7.41 (19.60–38.45)	27.6 ± 7.41 (19.19–32.35)	**0.02** ^3^
**Genetic subtype** **(Del/UPD/IC) ^1^**	19/13/1	11/13/1	0.99 ^5^
**BMI (kg/m^2^)** **(mean ± SD range)**	27.9 ± 5.70 (21.30–32.13) $\bar{x}$: [25.96, 29.84]	31.7 ± 9.66 (17.48–36.18) $\bar{x}$: [27.87, 35.53]	**0.03** ^4^
**BMI groups (1/2/3) ^2^** **No. (%)**	15/7/11 (45.4/21.2/33.3)	6/7/12 (24/28/48)	0.99 ^5^
**GH ^6^ Rx—past Rx** **No. (%)**	11 (33.3%)	15 (60%)	**0.04** ^3^
**GH current Rx** **No. (%)**	1 (3%)	1 (4%)	0.12 ^3^

^1^ Del, deletion; UPD, uniparental disomy; IC, imprinting center defects (microdeletion or epimutation). ^2^ BMI groups: group 1, below 25; group 2, between 25–30; group 3, 30 and above. ^3^ Using two-sided *t*-test. ^4^ Using one-sided *t*-test. ^5^ Using Pearson chi-square test. ^6^ GH, growth hormone. *p* values in bold indicate *p* < 0.05.

**Table 2:** Quality of Life Questionnaire (QOL) Scores.

QOL Domain	PWS’s Specialized Hostels (SH) n = 33 (Mean ± SD Range)	Home or Non-Specialized Hostels (H and NSH) n = 25 (Mean ± SD Range)	*p*-Value ^1^
**Physical Functioning**	90.0 ± 14.9	72.2 ± 26.8	**0.000**
**Role—Physical**	70.5 ± 39.3	67.0 ± 38.0	0.621
**Bodily Pain**	76.7 ± 25.9	80.2 ± 26.8	0.550
**General Health**	64.4 ± 20.8	71.0 ± 26.3	**0.037**
**Mental Health**	68.7 ± 14.9	73.3 ± 12.8	0.091
**Social Functioning**	74.2 ± 23.4	78.5 ± 32.0	0.291
**Role—Emotional**	66.7 ± 14.7	82.7 ± 33.5	0.068
**Vitality**	64.5 ± 17.5	57.8 ± 21.1	*p* = 0.464

^1^ all confounders including BMI groups: group 1, below 25; group 2, between 25–30; group 3, 30 and above. *p* values in bold indicate *p* < 0.05.

**Table 3:** Prader–Willi Syndrome Behavior Questionnaire (PWSBQ).

PWSBQ Domain	PWS’s Specialized Hostels (SH) n = 33 (Mean ± SD Range)	Home or Non-Specialized Hostels (H and NSH) n = 25 (Mean ± SD Range)	*p*-Value ^1^
**Abnormal emotional regulation**	10.4 ± 3.2	12.1 ± 4.2	*p* = 0.183
**Food-seeking-related behavior**	12.7 ± 9.7	18.5 ± 11.1	***p* = 0.039**
**Lack of flexibility**	15.2 ± 5.2	17.2 ± 7.0	*p* = 0.305
**Oppositional behavior and interpersonal problems**	9.7 ± 4.9	7.8 ± 6.0	***p* = 0.024**
**Total score**	47.9 ± 18.0	55.6 ± 24.1	*p* = 0.518

^1^ all confounders including BMI groups: group 1, below 25; group 2, between 25–30; group 3, 30 and above. *p* values in bold indicate *p* < 0.05.

## Data Availability

Data are unavailable due to privacy and ethical restrictions.

## Appendix A

### Appendix A.1. A Short Description of the PWS Specialized Group Homes in the Country

Each of the two group homes in the country operates in a similar manner. In the morning, residents attend work placements that are individually customized and closely supervised. The afternoons and evenings are structured with both therapeutic group activities, such as art therapy, dog therapy or bibliotherapy, and recreational activities. Daily physical exercise on a treadmill is mandatory for all residents, with each one having a preset time, speed, and incline determined by the dietitian. Strict “food security” measures are enforced throughout the day and night. All meals are meticulously planned by the dietitian, and residents are weighed several times a week. The staff comprises counselors covering all shifts, a social worker, and a visiting psychiatrist. Residents receive care from family doctors as needed and undergo annual follow-ups at the PWS Multi-Disciplinary Clinic within our medical center.

### Appendix A.2

**Table A1:** Comparison of the mean scores for quality of life questionnaire items between the years 2013 and 2021.

	QOL Domain:	PWS’s Specialized Hostels (SH)			Home or Non-Specialized Hostels (H and NSH)			Change Difference between SH and H and NSH (%)	*p *-Value
		n = 33			n = 25				
		2013	2021	Mean Change Difference (%)	2013	2021	Mean Change Difference (%)		
		(Mean ± SD Range)	(Mean ± SD Range)	(% ± SD Range)	(Mean ± SD Range)	(Mean ± SD Range)	(% ± SD Range)		
**physical domains**	**Physical Functioning**	51.7 ± 12.1	90 ± 14.9	**68.4 ± 0.3**	49.6 ± 16.1	72.2 ± 26.8	**46.58 ± 0.24**	21.82%	0.07
	**Role—Physical**	47.07 ± 13.9	70.4 ± 39.2	**56.13 ± 1.13**	46.6 ± 15.9	67 ± 38	**6.49 ± 0.84**	49.64%	0.247
	**Bodily Pain**	47.03 ± 16.9	76.7 ± 25.9	**65.8 ± 0.63**	46.4 ± 20.1	80.2 ± 26.8	**46.5 ± 0.73**	19.32%	0.47
	**General Health**	48.4 ± 15.6	64.4 ± 20.8	**35 ± 0.6**	48.2 ± 19.3	71 ± 26.7	**33. ± 0.33**	1.68%	0.934
**mental domains**	**Mental Health**	44.8 ± 15.6	68 ± 14.08	**63.1 ± 0.5**	468 ± 17.1	73.3 ± 12.8	**51.5 ± 0.38**	11.65%	0.546
	**Social Functioning**	42.02 ± 14.6	74. ± 23.4	**87.05 ± 0.74**	43.5 ± 17.2	78.5 ± 32	**76 ± 0.8**	10.62%	0.731
	**Role—Emotional**	40.9 ± 15.7	667 ± 41.7	**56.4 ± 0.9**	45.08 ± 17.02	82.7 ± 33.5	**87.4 ± 0.9**	31.06%	0.391
	**Vitality**	493 ± 15.8	6 ± 17.5	**37.4 ± 0.4**	49.3 ± 17.9	57.8 ± 21.1	**18.7 ± 0.3**	19.07%	0.201

### Appendix A.3

**Table A2:** Comparison of the mean scores from behavior questionnaires between 2013 and 2021.

PWSBQ Domain	PWS’s Specialized Hostels (SH) n = 33			Home or Non-Specialized Hostels (H and NSH) n = 25			Change Difference between SH and H and NSH (%)	*p*-Value
	2013	2021	Mean Change Difference (%)	2013	2021	Mean Change Difference (%)		
	(Mean ± SD)	(Mean ± SD)	(% ± SD)	(Mean ± SD)	(Mean ± SD)	(% ± SD)		
**Abnormal emotional regulation**	11.04 ± 4.5	10.6 ± 5	**−2.79 ± 0.42**	10.6 ± 5	12.1 ± 4.2	**15.05 ± 0.5**	−18%	0.221
**Food-seeking-related behavior**	19.4 ± 10.4	17.3 ± 10.6	**−41.57 ± 0.5**	17.3 ± 10.6	18.5 ± 11.1	**22.78 ± 0.75**	**−64%**	**0.003**
								**β: [−110.10%,** **−18.60%]**
**Lack of flexibility**	16.4 ± 5.8	15.2 ± 5.2	**−5.35 ± 0.5**	17.5 ± 6.6	17.2 ± 7.0	**−8.12 ± 0.5**	3%	0.871
**Oppositional behavior and** **interpersonal problems**	11.3 ± 5.8	17.5 ± 6.6	**8.7 ± 1.4**	8.89 ± 6.2	7.8 ± 6.0	**17.43 ± 1.2**	−9%	0.848
**Total score**	58.2 ± 19.7	8.89 ± 6.2	**−15.7 ± 0.5**	54.3 ± 24.05	55.6 ± 24.1	**0.29 ± 0.45**	−16%	0.34

### Appendix A.4. Full List of Medical Conditions Found in the Sample

Endocrine and Metabolic Disorders:

- Hypothyroidism;
- Hypogonadism;
- Impaired fasting glycemia (IFG);
- Diabetes mellitus;
- Diabetes insipidus;
- Hypercholesterolemia;
- Hypertriglyceridemia;
- Iron-deficiency anemia;
- Other types of anemia;
- Hyperprolactinemia;
- Vitamin deficiency (folic acid, B12, vitamin D).

Cardiovascular Conditions:

- Hypertension;
- Innocent heart murmurs;
- Deep vein thrombosis (DVT);
- Pulmonary embolism.

Respiratory Disorders:

- Sleep apnea;
- Asthma;
- Lung abscess.

Neurological Conditions:

- Narcolepsy;
- Attention-deficit/hyperactivity disorder (ADHD);
- Convulsions;
- Gaze tonic upward paroxysmal.

Ophthalmic Conditions:

- Diabetic retinopathy;
- Strabismus;
- Myopia;
- Astigmatism;
- Keratoconus;
- Hypermetropia;
- Esotropia.

Digestive System Conditions:

- Chronic pancreatitis;
- Diarrhea;
- Anal fissure;
- Rectal prolapse;
- Hemorrhoids;
- Colectomy.

Musculoskeletal Disorders:

- Fractures;
- Fixation surgery;
- Kyphosis;
- Scoliosis;
- Osteopenia;
- Osteoporosis.

Dermatological Conditions:

- Dermatofibroma;
- Atopic dermatitis.

Urogenital Conditions:

- Lymphedema;
- Cryptorchidism;
- Orchiopexy;
- Ovarian cyst;
- Dysuria;
- Anuresis;
- Oligomenorrhea;
- Amenorrhea;
- Gynecomastia;
- Hernia;
- Inguinal hernia.

Infectious Diseases:

- COVID-19.

Others:

- Diabetic nephropathy (listed twice, suggesting confirmation);
- Elevated liver enzymes;
- Calcinosis;
- Hypernatremia.

### Appendix A.5

**Table A3:** Mean blood test results divided by research groups.

		PWS’s Specialized Hostels n = 33	Non-Specialized Hostels/ Home Group n = 25	Mean Duration of Stay in Specialized Hostel (Year)	*p*-Value
	Units	(Mean ± SD Range)	(Mean ± SD Range)		
Sugar profile:					
Glucose (B)	mg/dL	90 ± 36.4	92.8 ± 31.3	8.37	0.281
HB A1C (%)	%	5.5 ± 1.6	5.8 ± 1.7	8.99	0.117
Lipid profile:					
Cholesterol	mg/dL	166.9 ± 33.1	175.2 ± 30.8	8.39	0.001
LDL Cholesterol	mg/dL	100.3 ± 29.5	107.8 ± 28.5	8.42	0.002
HDL Cholesterol	mg/dL	49.3 ± 11.98	46.9 ± 9.3	8.46	0.008
Triglycerides	mg/dL	80.8 ± 38.8	102.9 ± 49.8	8.4	0
Vitamin D (25-OH)	ng/mL	33.2 ± 10.2	26.9 ± 10.3	8.35	0
Parameters to evaluate the risk of metabolic syndrome:					
Triglyceride/HDL ratio	ratio	1.7 ± 1.02	2.3 ± 1.3	8.44	0
Triglyceride glucose index	ratio	8.08 ± 0.54	8.3 ± 0.6	8.48	0.013
